# Incidence, Risk Factors, and Management of Conjunctivitis in Atopic Dermatitis Patients Treated With Dupilumab or Tralokinumab: Results From a Multicenter, Observational, Retrospective Study

**DOI:** 10.1111/ijd.70027

**Published:** 2025-08-20

**Authors:** Luca Potestio, Cataldo Patruno, Marika Del Gaudio, Michela Ortoncelli, Simone Ribero, Francesca Barei, Silvia Mariel Ferrucci, Laura Bonzano, Ilaria Trave, Mario Bruno Guanti, Carlotta Gurioli, Bianca Maria Piraccini, Niccolò Gori, Elena Ippoliti, Ketty Peris, Laura Grigolato, Mariateresa Rossi, Caterina Foti, Paolo Romita, Benedetta Tirone, Eustachio Nettis, Maria Esposito, Maria Concetta Fargnoli, Lina Maria Magnanimi, Elena Pezzolo, Katharina Hansel, Luca Stingeni, Rosanna Satta, Maria Mariano, Flavia Pigliacelli, Flavia Manzo Margiotta, Marco Romanelli, Ruggero Cascio Ingurgio, Antonio Costanzo, Alessandra Narcisi, Monica Corazza, Emiliano Antiga, Pietro Morrone, Pietro Rubegni, Laura Calabrese, Laura Lazzeri, Giovanni Rubegni, Fabrizio Guarneri, Nicola Zerbinati, Andrea Carugno, Rosa Valentina Puca, Oriele Sarno, Claudio Marasca, Emanuela Martina, Francesca di Vico, Maddalena Napolitano

**Affiliations:** ^1^ Section of Dermatology, Department of Clinical Medicine and Surgery University of Naples Federico II Naples Italy; ^2^ Department of Medicine and Health Sciences “Vincenzo Tiberio” University of Molise Campobasso Italy; ^3^ Department of Medical Sciences, Dermatology Clinic University of Turin Turin Italy; ^4^ Dermatology Unit Fondazione IRCCS Ca' Granda Ospedale Maggiore Policlinico Milan Italy; ^5^ Dermatology Unit Arcispedale Santa Maria Nuova, Azienda USL‐IRCCS Reggio Emilia Reggio Emilia Italy; ^6^ Section of Dermatology, Department of Health Sciences (DISSAL) University of Genoa, IRCCS Ospedale Policlinico San Martino Genoa Italy; ^7^ Department of Dermatology University of Modena and Reggio Emilia Modena Italy; ^8^ Dermatology Unit IRCCS Azienda Ospedaliero‐Universitaria di Bologna Bologna Italy; ^9^ Department of Medical and Surgical Sciences Alma Mater Studiorum, University of Bologna Bologna Italy; ^10^ Dermatology, University Department of Translational Medicine and Surgery Sacred Heart Catholic University Rome Italy; ^11^ Unit of Dermatology, Department of Medical and Surgical Sciences IRCCS A. Gemelli University Polyclinic Foundation Rome Italy; ^12^ Dermatology Unit University of Brescia, ASST Spedali Civili of Brescia Brescia Italy; ^13^ Section of Dermatology and Venereology, Department of Precision and Regenerative Medicine and Ionian Area (DiMePRe‐J) University of Bari “Aldo Moro” Bari Italy; ^14^ Regional Reference Center for Allergic and Immunological Diseases, Department of Precision and Regenerative Medicine and Ionian Area (DiMePRe‐J) University of Bari “Aldo Moro” Bari Italy; ^15^ Department of Biotechnological and Applied Clinical Sciences University of L' Aquila L' Aquila Italy; ^16^ Department of Dermatology San Bortolo Hospital Vicenza Italy; ^17^ Dermatology Section, Department of Medicine and Surgery University of Perugia Perugia Italy; ^18^ Department of Medical, Surgical, and Experimental Sciences University of Sassari Sassari Italy; ^19^ Clinical Dermatology San Gallicano Dermatological Institute, IRCCS Rome Italy; ^20^ Department of Dermatology University of Pisa Pisa Italy; ^21^ Health Science Interdisciplinary Center Sant'Anna School of Advanced Studies Pisa Italy; ^22^ Dermatology Unit IRCCS Humanitas Research Hospital Milan Italy; ^23^ Department of Biomedical Sciences Humanitas University Milan Italy; ^24^ Section of Dermatology, Department of Medical Sciences University of Ferrara Ferrara Italy; ^25^ Section Dermatology, Department of Health Sciences University of Florence Florence Italy; ^26^ Dermatology Azienda Provinciale di Cosenza Cosenza Italy; ^27^ Dermatology Unit, Department of Medical, Surgical and Neurological Sciences University of Siena Siena Italy; ^28^ Institute of Dermatology Sacred Heart Catholic University Rome Italy; ^29^ Ophthalmology Unit, Department of Medicine, Surgery and Neurosciences University of Siena Siena Italy; ^30^ Department of Clinical & Experimental Medicine, Section of Dermatology University of Messina Messina Italy; ^31^ Department of Medicine and Innovation Technology (DiMIT) University of Insubria Varese Italy; ^32^ Department of Medicine and Surgery University of Insubria Varese Italy; ^33^ Department of Dermatology and Dermosurgery AOSG San Giuseppe Moscati Avellino Italy; ^34^ Dermatology Unit, Medical Department National Hospital “A. Cardarelli” of Naples Naples Italy; ^35^ Dermatology Clinic, Department of Clinical and Molecular Sciences Polytechnic Marche University Ancona Italy

## Abstract

**Background:**

Conjunctivitis is among the most frequent adverse events (AEs) emerged in clinical trials for all biologic drugs approved for atopic dermatitis (AD). However, real‐world comparative data on the incidence, risk factors, and management of conjunctivitis remain limited.

**Objective:**

We aimed to compare the incidence, clinical features, and management of conjunctivitis in patients with moderate‐to‐severe AD treated with dupilumab or tralokinumab in a real‐life setting.

**Patients and Methods:**

A multicenter, retrospective, observational study including adult patients with moderate‐to‐severe AD treated with dupilumab or tralokinumab for at least 16 weeks was carried out. Demographic, clinical, and therapeutic data were collected from 35 dermatological referral centers across Italy. Conjunctivitis incidence, severity, time to onset, and ophthalmologic management were analyzed and compared between treatment groups.

**Results:**

A total of 6668 patients were included (5899 on dupilumab and 769 on tralokinumab). Conjunctivitis occurred in 10.76% of dupilumab‐treated and 12.61% of tralokinumab‐treated patients, with no statistically significant difference in overall incidence. However, time to onset was significantly shorter with tralokinumab than with dupilumab (15.3 ± 14.5 weeks vs. 35.5 ± 45.2 weeks, respectively; *p* < 0.0001). Ophthalmologic management strategies were similar between groups, mainly involving lubricants and corticosteroid‐based eye drops. Dupilumab‐treated patients more frequently discontinued or switched treatment due to conjunctivitis than tralokinumab patients (25.4% vs. 14.4%, respectively; *p* < 0.05).

**Conclusions:**

Conjunctivitis represents a relatively frequent AE in patients with AD receiving dupilumab or tralokinumab. However, earlier onset of conjunctivitis with tralokinumab and higher discontinuation rates with dupilumab suggest differing tolerability profiles. Early recognition of ocular symptoms is essential, and dermatologists should promptly initiate supportive eye care and refer to ophthalmologists when appropriate to avoid unnecessary treatment interruptions.

**Key points**

- This retrospective real‐life multicenter study compared the incidence, risk factors, and management of conjunctivitis in patients with moderate‐to‐severe atopic dermatitis treated with dupilumab or tralokinumab.
- While the overall incidence of conjunctivitis was comparable between treatment groups, tralokinumab was associated with an earlier onset, whereas dupilumab led more frequently to treatment discontinuation or switching due to ocular adverse events.
- Our findings underscore the need for early recognition and proactive management of conjunctivitis in patients affected by atopic dermatitis receiving biologics to optimize treatment adherence and outcomes.

## 1 Introduction

Atopic dermatitis (AD) is a chronic, relapsing inflammatory skin disease with a significant impact on quality of life [1, 2]. It often coexists with other atopic conditions, including asthma, rhinitis, and conjunctivitis [3]. AD pathogenesis involves genetic and environmental factors, skin barrier dysfunction, *Staphylococcus aureus* colonization, and a predominant T helper 2 (Th2) immune response [4, 5, 6, 7]. Central to this process are interleukins (IL)‐4 and IL‐13, which impair barrier integrity, activate eosinophils and mast cells, and promote IgE production [8, 9, 10, 11]. These cytokines also contribute to pruritus and ocular surface inflammation, making them key targets for modern biologic therapies [10, 11]. These insights have led to the development of targeted biologics that block IL‐4 and/or IL‐13 pathways, significantly advancing the treatment landscape for moderate‐to‐severe AD [12].

Currently, three biologics are approved for AD: dupilumab, tralokinumab, and lebrikizumab. Dupilumab is a fully human monoclonal antibody that inhibits both IL‐4 and IL‐13 signaling by targeting the shared IL‐4 receptor alpha subunit [13]. It is approved by the United States Food and Drug Administration (FDA) and the European Medicines Agency (EMA) for patients aged ≥ 6 months. Tralokinumab is a high‐affinity IL‐13–neutralizing antibody approved for adults and adolescents aged ≥ 12 years [14, 15]. Lebrikizumab, a recently approved IL‐13–targeting antibody for patients aged ≥ 12 years, differs from tralokinumab by preventing IL‐13 from binding to the IL‐4Rα/IL‐13Rα1 receptor complex, rather than both IL‐13Rα1 and IL‐13Rα2 [16, 17].

Clinical trials have demonstrated the efficacy and safety of dupilumab [18, 19, 20], tralokinumab [21, 22], and lebrikizumab [16, 23] in the treatment of AD. Conjunctivitis is among the most frequent adverse events (AEs) for all these drugs. A systematic review and meta‐analysis including 40 randomized controlled trials, involving 14,598 participants, provided data on the impact of treatment on conjunctivitis incidence. Moderate‐to‐high certainty evidence showed that dupilumab (odds ratio [OR] 2.88 [95% confidence interval [CI] 2.13–3.90]; risk difference: 40 additional cases per 1000 patients [95% CI 25–61]), lebrikizumab (OR 2.58 [95% CI 1.33–4.99]; risk difference: 34 additional cases per 1000 [95% CI 7–82]), and tralokinumab (OR 2.46 [95% CI 1.60–3.77]; risk difference: 32 additional cases per 1000 [95% CI 13–58]) were similarly associated with an increased risk of conjunctivitis. Other agents either showed no significant difference compared to placebo or were supported by lower certainty evidence [24].

Proposed mechanisms for this AE include reduced goblet cell density due to IL‐13 inhibition, altered mucin production, increased *Demodex* mite density, and immune dysregulation in conjunctival‐associated lymphoid tissue, frequent eye rubbing in AD subjects which acts as a mechanical and inflammatory amplifier in an already vulnerable ocular environment (patients with blepharitis and/or conjunctivitis before starting treatment) [25, 26, 27, 28].

Standardized approaches to prevent conjunctivitis in patients receiving biologics are lacking, and treatments often rely on the use of ophthalmic preparations containing lubricants, corticosteroids, antibiotics, or topical calcineurin inhibitors. A 2019 consensus from the International Eczema Council highlighted the importance of early identification and management of conjunctivitis in AD patients treated with dupilumab by screening all the patients for ocular symptoms and informing them of the risk of conjunctivitis prior to initiating dupilumab [29]. Importantly, a history of conjunctivitis should not preclude dupilumab use, and treatment should generally be continued even in the event of new‐onset conjunctivitis, with timely referral to an ophthalmologist [29]. Dermatologists are encouraged to initiate basic ocular management (e.g., lubricating drops or antihistamines) in case of dupilumab‐associated conjunctivitis, but the use of corticosteroid or immunomodulatory eye drops should be guided by ophthalmologists [29].

Despite the increasing use of biologics for AD, as well as increasing real‐life data studies on their effectiveness and safety, comparative real‐world data on conjunctivitis incidence, risk factors, and management between dupilumab and tralokinumab remain limited [30].

This multicenter, real‐life study aimed to compare the incidence of conjunctivitis in AD patients treated with dupilumab or tralokinumab, identify potential risk factors, and describe real‐world management strategies. Lebrikizumab has been excluded from our study since its recent approval limits the availability of real‐life data.

## 2 Material and Methods

A multicenter, real‐life, retrospective, observational study was conducted involving patients affected by moderate‐to‐severe confirmed diagnosis of AD who had been receiving treatment with either dupilumab or tralokinumab for a minimum duration of 16 weeks. Data were collected from 35 academic and nonacademic dermatological referral centers evenly distributed throughout Northern, Central, and Southern Italy.

Eligible participants met the following inclusion criteria: age ≥ 18 years, a confirmed diagnosis of AD established by a dermatologist, and ongoing treatment with dupilumab or tralokinumab for at least 16 weeks at the time of data collection. The following demographic and clinical parameters were recorded for all included patients: age, sex, age at disease onset, disease duration, biologic treatment initiation date, atopic comorbidities (asthma, rhinitis, conjunctivitis, eosinophilic esophagitis), and history of prior systemic therapies for AD. No standardized wash‐out period was applied between prior systemic therapies and biologic initiation, reflecting routine clinical practice in a real‐world setting. For patients who developed conjunctivitis during treatment, detailed clinical information was gathered, including a previous history of conjunctivitis, severity of ocular involvement (mild, moderate, severe), any ophthalmologic diagnosis if available, time between biologic initiation and conjunctivitis onset or worsening of a previous conjunctivitis, referral to ophthalmologic care, and therapeutic management, including both ocular and biologic treatment decisions. Ocular severity was categorized as mild, moderate, or severe based on clinical judgment at each center. Mild cases typically involved limited conjunctival hyperemia or discomfort without requiring ophthalmologic referral. Moderate cases included more persistent symptoms (e.g., tearing, burning, or foreign body sensation) often requiring topical treatment. Severe cases involved marked symptoms impacting quality of life or necessitating referral to an ophthalmologist and/or changes in biologic treatment. Patients were analyzed according to the initial biologic therapy under which conjunctivitis occurred. Those who switched to another biologic were not reclassified into the new treatment cohort. Conjunctivitis was defined as the occurrence of ocular signs or symptoms suggestive of conjunctival inflammation (e.g., redness, tearing, pruritus, discomfort), as documented by the treating dermatologist and/or ophthalmologist. Diagnosis was based on clinical judgment and, when applicable, the initiation of topical ocular treatment. No standardized diagnostic protocol was applied across centers.

Continuous variables were reported as mean ± standard deviation (SD), whereas categorical variables were summarized as absolute frequencies and corresponding percentages. Comparisons between the dupilumab and tralokinumab cohorts were conducted using Student's *t*‐test for continuous variables and the Chi‐square test for categorical variables. Finally, an ordinal logistic regression was performed to assess the association between clinical factors and the severity of conjunctivitis, using a proportional odds model with a logit link function. Severity was treated as an ordinal outcome (1 = mild, 2 = moderate, 3 = severe).

Independent variables included sex, age, history of conjunctivitis, and the time interval (in weeks) between initiation of biologic drugs and onset of conjunctivitis. Model assumptions were verified; statistical significance was set at *p* < 0.05. All statistical analyses were performed using GraphPad Prism software, version 8.0 (GraphPad Software Inc., La Jolla, CA, USA), with statistical significance defined as *p* < 0.05.

The study complied with the Declaration of Helsinki and local ethical regulations. As it involved anonymized retrospective data, formal ethical approval was waived.

## 3 Results

A total of 6668 patients met the inclusion and exclusion criteria of the study. Specifically, 5899 (88.5%) patients (3130 males [53.1%]; mean age 40.6 ± 21.7 years) were treated with dupilumab, whereas 769 (11.5%) subjects (455 males [59.2%]; mean age 44.4 ± 21.1 years) received tralokinumab. Baseline demographic and clinical characteristics are summarized in Table 1.

**TABLE 1:** Demographic and clinical features of patients treated with dupilumab or tralokinumab.

Study population			
	Dupilumab	Tralokinumab	*p*
Number of patients	5899	769	< 0.0001
Sex, male	3130 (53.1%)	455 (59.2%)	< 0.01
Mean age	40.6 ± 21.7	44.4 ± 21.1	< 0.0001
Age of AD onset (years)	19.2 ± 23.4	24.0 ± 23.4	< 0.0001
Mean duration of AD (years)	22.3 ± 17.5	20.3 ± 17.3	< 0.01
Atopic comorbidities			
Rhinitis	2342 (39.7%)	342 (44.5%)	< 0.05
Asthma	1522 (25.8%)	200 (26.0%)	ns
Conjunctivitis	1585 (26.9%)	214 (27.8%)	ns
Eosinophilic esophagitis	69 (1.2%)	5 (0.7%)	ns
Previous systemic treatment^a^			
Oral corticosteroids	4708 (79.8%)	631 (82.1%)	ns
Cyclosporine	3609 (61.2%)	523 (68.0%)	< 0.001
Methotrexate	183 (3.1%)	31 (4.0%)	ns
Phototherapy	838 (14.2%)	124 (16.1%)	ns
Dupilumab	0 (0%)	170 (22.1%)	na
Tralokinumab	75 (1.3%)	0 (0%)	na
Abrocitinib	19 (0.3%)	3 (0.4%)	ns
Baricitinib	16 (0.3%)	7 (0.9%)	< 0.01
Upadacitinib	57 (1.0%)	39 (5.1%)	< 0.0001
Naïve to biologics/JAK inhibitors	5741 (97.3%)	571 (74.3%)	< 0.0001

Abbreviations: AD, atopic dermatitis; na, not applicable; ns, not significant.

^a^ All patients had received more than one prior systemic treatment before initiating biologic therapy with dupilumab or tralokinumab.

The tralokinumab cohort had a significantly higher mean age (*p* < 0.0001), later disease onset (24.0 ± 23.4 vs. 19.2 ± 23.4 years, *p* < 0.0001), and a greater proportion of male patients (*p* < 0.01) compared to the dupilumab group. Conversely, the dupilumab group had a significantly longer disease duration (22.3 ± 17.5 vs. 20.3 ± 17.3 years, *p* < 0.01). Atopic comorbidities were largely comparable between groups, with the exception of rhinitis, which was slightly more prevalent in the tralokinumab group (44.5% vs. 39.7%, *p* < 0.05). The prevalence of prior conjunctivitis was similar.

Concerning previous systemic treatments, patients on tralokinumab had been more frequently treated with cyclosporine (68.0% vs. 61.2%; *p* < 0.001), baricitinib (0.9% vs. 0.3%; *p* < 0.01), or upadacitinib (5.1% vs. 1.0%; *p* < 0.0001). Conversely, a significantly higher proportion of dupilumab patients were biologic/Janus kinase (JAK) inhibitor‐naïve (97.3% vs. 74.3%, *p* < 0.0001), indicating a wider prior exposure to immunomodulators in the tralokinumab cohort.

Conjunctivitis occurred in 635 of 5899 (10.8%) dupilumab‐treated patients and in 97 of 769 (12.6%) tralokinumab‐treated patients. Clinical features of affected patients are detailed in Table 2.

**TABLE 2:** Clinical characteristics of patients treated with dupilumab or tralokinumab developing conjunctivitis, including previous history of conjunctivitis, conjunctivitis severity, and management.

Patients developing conjunctivitis			
	Dupilumab	Tralokinumab	*p*
Number of patients	635/5899 (10.8%)	97/769 (12.6%)	ns
Sex, male	339 (53.4%)	42 (43.3%)	ns
Mean age (years)	37.4 ± 15.5	38.7 ± 16.3	ns
History of conjunctivitis	288 (45.4%)	47 (48.5%)	ns
Severity of conjunctivitis			
Mild	263 (41.4%)	48 (49.5%)	ns
Moderate	164 (25.8%)	21 (21.7%)	ns
Severe	208 (32.8%)	28 (28.9%)	ns
Time (weeks)^a^	35.5 ± 45.2	15.3 ± 14.5	< 0.0001
Ophthalmologist referrals	255 (40.3%)	29 (29.9%)	ns
Ophthalmologic treatment			
Moisturizing eye drops	303 (47.7%)	48 (49.5%)	ns
Corticosteroids eye drops	147 (23.2%)	23 (23.7%)	ns
Corticosteroids + antibiotics eye drops	108 (17.0%)	14 (14.4%)	ns
Cyclosporine eye drops	53 (8.4%)	8 (8.3%)	ns
Topical calcineurin inhibitors	24 (3.8%)	4 (4.1%)	ns
Biologic treatment management			
No treatment interruption	474 (74.7%)	83 (85.6%)	< 0.05
Switch to dupilumab	0 (0%)	3 (3.1%)	na
Switch to tralokinumab	33 (5.2%)	0 (0%)	na
Switch to lebrikizumab	10 (1.6%)	1 (1.0%)	ns
Switch to abrocitinib	13 (2.1%)	2 (2.1%)	ns
Switch to baricitinib	19 (3.0%)	2 (2.1%)	ns
Switch to upadacitinib	86 (13.5%)	6 (6.2%)	< 0.05

Abbreviations: na, not applicable; ns, not significant.

^a^ Time between biologic treatment initiation and onset of conjunctivitis.

Although the overall incidence was slightly higher in the tralokinumab group, this difference was not statistically significant.

Interestingly, the mean time to onset of conjunctivitis was significantly shorter in tralokinumab‐treated patients (15.3 ± 14.5 weeks) compared to those treated with dupilumab (35.5 ± 45.2 weeks, *p* < 0.0001). While the dupilumab group showed a trend toward more frequent severe conjunctivitis and ophthalmologic referrals, none of these differences reached statistical significance.

In terms of ophthalmologic management, the most frequently used treatments were moisturizing eye drops (dupilumab: 303 [47.7%]; tralokinumab: 48 [49.5%]), corticosteroid‐containing eye drops (dupilumab: 147 [23.2%]; tralokinumab: 23 [23.7%]), and combination corticosteroid‐antibiotic preparations (dupilumab: 108 [17.0%]; tralokinumab: 14 [14.4%]), with no significant differences between the two groups. Cyclosporine (dupilumab: 53 [8.4%]; tralokinumab: 8 [8.3%]) and topical calcineurin inhibitors (dupilumab: 24 [3.8%]; tralokinumab: 4 [4.1%]) were prescribed in a minority of cases.

Regarding treatment continuation, a significantly lower proportion of patients in the dupilumab group continued treatment without interruption due to conjunctivitis (74.7% vs. 85.6%; *p* < 0.05). Consequently, switches to other biologics or JAK inhibitors were more frequent among dupilumab‐treated patients, particularly to upadacitinib (13.5% vs. 6.2%, *p* < 0.05).

Notably, a total of 33 dupilumab patients (5.2%) switched to tralokinumab and 10 (1.6%) to lebrikizumab, whereas 3 (3.1%) and 1 (1.0%) tralokinumab patients later received dupilumab or lebrikizumab, respectively. In all these cases, an improvement in conjunctivitis was observed. Of note, all treatment switches were motivated by the occurrence of conjunctivitis.

Subgroup analysis showed no significant associations between sex, prior conjunctivitis history, or biologic type and conjunctivitis onset. Ordinal logistic regression in the dupilumab cohort did not identify any statistically significant predictors of conjunctivitis severity. Among tested variables (sex, age, history of conjunctivitis, and the time interval [in weeks] between initiation of biologic drugs and onset of conjunctivitis, prior conjunctivitis, and time to symptom onset), male sex showed a nonsignificant trend toward more severe conjunctivitis (*p* = 0.097). No significant predictors were identified in the tralokinumab cohort.

## 4 Discussion

Conjunctivitis represents one of the most frequently reported and clinically impactful AEs in patients with AD treated with dupilumab or tralokinumab. Although generally mild to moderate, its occurrence can lead to discomfort, impaired quality of life, and in some cases, treatment discontinuation or switching [30, 31]. As targeted AD therapies become increasingly widespread, a deeper understanding of conjunctivitis risk, timing, and management is essential in daily clinical practice [31, 32].

A pooled analysis of 11 randomized, double‐blinded, placebo‐controlled phase II and III trials investigating dupilumab in AD (*n* = 6, 2629 patients), asthma (*n* = 3, 2876 patients), chronic rhinosinusitis with nasal polyps (*n* = 1, 60 patients), and eosinophilic esophagitis (*n* = 1, 47 patients) showed a higher incidence of conjunctivitis in dupilumab‐treated patients with AD compared to placebo (8.6%–22.1% vs. 2.1%–11.1%). Moreover, greater disease severity and a prior history of conjunctivitis were associated with an increased risk of developing conjunctivitis [33]. Of note, most cases were mild to moderate, resolving in 80% of subjects while continuing dupilumab treatment, and leading to permanent interruption only in two patients. Furthermore, the incidence of conjunctivitis in trials investigating the use of dupilumab for type two conditions other than AD was lower for both dupilumab and placebo compared to the AD trial [33]. As a consequence, the risk of conjunctivitis has been suggested to be specifically related to the use of dupilumab in AD management [33].

A recent multicenter retrospective study by Reguiai et al., investigating the characteristics of patients who developed conjunctivitis requiring discontinuation of dupilumab (83 subjects registered, data available for 71 patients), reported a mean time of conjunctivitis onset following biologic initiation of 4.5 ± 3.63 months [34]. Furthermore, a multivariate analysis showed that longer conjunctivitis duration, personal history of asthma, and switching from dupilumab to JAK inhibitors were the only factors significantly associated with a complete resolution of dupilumab‐associated conjunctivitis (*p* = 0.018, *p* = 0.009, and *p* = 0.002, respectively) [34]. Differently, in our study, a mean time of conjunctivitis onset following biologic initiation of 29.0 ± 41.4 weeks was registered in patients who interrupted treatment with dupilumab. Furthermore, no predictive factors for complete resolution of conjunctivitis were identified.

In a large multicenter retrospective cohort study enrolling 210 patients, [35] et al. reported a 37% (*n* = 78) incidence of dupilumab‐induced ocular surface disease (DIOSD) among dupilumab‐treated AD patients over 52 weeks, with most cases presenting as blepharoconjunctivitis and requiring topical management [35]. Treatment discontinuation due to DIOSD occurred in only 4% of patients, highlighting the importance of early recognition and intervention. Compared to our study, the higher incidence (37% vs. 10.8%) may reflect the more focused evaluation of ocular symptoms, while the lower discontinuation (4% vs. 25.3%) rate suggests that timely management may prevent treatment interruption. Recent data from the BioDay registry, involving 1223 subjects treated with dupilumab for up to 5 years, reported conjunctivitis as the most common AE (35.2% of adults and 19.1% of pediatric patients), leading to discontinuation in 42 (3.4%) cases [36]. Finally, in a prospective study of 69 dupilumab‐treated AD patients, Achten et al. reported that 20 (28.9%) developed DIOSD after treatment initiation, despite 91% already showing ocular surface abnormalities at baseline. Ophthalmologic assessments were standardized using the UTOPIA score, and cytologic analysis showed a decrease in CK19‐CD45‐MUC5AC+ cells, suggesting impaired goblet cell function. Early ophthalmic treatment appeared to reduce DIOSD severity over time [37].

As regards tralokinumab, data from five randomized, placebo‐controlled trials including 2285 AD subjects reported an incidence of mainly mild‐to‐moderate conjunctivitis higher (7.5%) than placebo (3.2%) [37]. Of note, 78.6% of conjunctivitis resolved during the trials, and only two patients permanently discontinued tralokinumab. AD severity at baseline, history of allergic conjunctivitis/atopic keratoconjunctivitis, and presence of atopic comorbidities were associated with an increased risk of conjunctivitis [38].

In the recent real‐world study by Dekkers et al. involving 84 patients treated with tralokinumab, 16 cases of conjunctivitis (19.0%) were reported, leading to treatment discontinuation in 5/16 patients (31.3%) [39]. In comparison, our study showed a lower incidence (12.6%) and a lower discontinuation rate (14.4%). These differences may be explained by the prospective design of Dekkers et al., which allowed for better capture of mild cases, while our retrospective approach may have missed such events. Conversely, the different discontinuation rate in our cohort may reflect differences in clinical practice and greater access to alternative therapies [39].

Lastly, a systematic review and meta‐analysis investigating the incidence of conjunctivitis AE in patients treated with biologics for AD in clinical trials (17 studies: 12 dupilumab, 2 lebrikizumab, and 3 tralokinumab) found that among 4197 patients undergoing biologic therapies, 213 (5.08%) reported conjunctivitis as compared to 32 out of 1633 (1.96%) participants in the control group (receiving placebo) [30]. Interestingly, no significant differences were found between subgroups treated with different agents (*p* = 0.89) [30].

In our multicenter, real‐world study involving over 6600 patients, we investigated the incidence and characteristics of conjunctivitis in patients treated with dupilumab or tralokinumab. We found that conjunctivitis occurred in approximately 11%–13% of patients, with no significant difference in overall incidence between the two treatment groups. However, important distinctions emerged in terms of timing and therapeutic consequences. Conjunctivitis appeared significantly earlier in patients receiving tralokinumab, with a mean onset of just over 15 weeks compared to 35 weeks in the dupilumab group. While the exact reason for this difference remains unclear, it suggests potential early ocular surface vulnerability associated with selective IL‐13 inhibition. Despite the earlier onset of conjunctivitis with tralokinumab, patients on dupilumab were more likely to discontinue or switch treatment due to ocular side effects, highlighting a greater clinical impact in this group. In our real‐world cohort, clinical severity and ophthalmologic management strategies were largely similar between groups, typically involving lubricating drops and corticosteroid‐based therapies.

Overall, conjunctivitis was a relatively frequent AE in both treatment groups, with largely comparable clinical severity and management strategies. However, the earlier conjunctivitis onset observed with tralokinumab and the higher treatment discontinuation rate observed with dupilumab suggest possible differences in tolerability profiles that warrant further investigation.

Our study benefits from a large, diverse patient population and a real‐world setting, capturing practical management decisions not typically reflected in controlled trials. Interestingly, since the study involved multiple centers uniformly distributed across northern, central, and southern Italy, the cohort was unlikely to be affected by local pollen variability. Therefore, the reported drug‐related conjunctivitis cases were not influenced by this confounding factor. Moreover, the large sample size further minimizes the risk of bias related to seasonal or perennial allergen exposure. This study has several limitations. First, its retrospective design and the sample size imbalance between dupilumab and tralokinumab groups may have introduced bias. The lack of AD severity data could also affect the interpretation of conjunctivitis risk and treatment decisions. Since the primary aim was to evaluate ocular outcomes rather than efficacy, disease severity was not systematically assessed, which remains a possible confounding factor. In addition, ophthalmologic evaluations were not standardized across centers, limiting detailed characterization of ocular findings. We were also unable to retrieve consistent data on patients who discontinued biologic therapy before reaching 16 weeks, which may have led to an underestimation of early‐onset conjunctivitis. No wash‐out period was applied between prior treatments and biologic initiation, raising the possibility that ocular AEs from previous therapies may have overlapped with those observed during the current treatment. Finally, lebrikizumab was not included in our analysis due to its recent approval and limited use in clinical practice during the data collection period.

To sum up, conjunctivitis continues to represent a common and clinically significant AE in patients with AD receiving biologic therapies, with notable differences observed between dupilumab and tralokinumab in terms of onset and clinical management. Early recognition of ocular symptoms is essential, and dermatologists should promptly initiate supportive eye care and refer to ophthalmologists when appropriate. Further prospective research is warranted to elucidate underlying mechanisms, identify predictive risk factors, and establish evidence‐based strategies for prevention and treatment.

## Ethics Statement

Ethical approval was not required, as the study involved retrospective data collection without deviation from routine clinical care. All participants had provided written informed consent for the use of their anonymized clinical data during standard clinical visits. The study complied with the Declaration of Helsinki.

## Conflicts of Interest

The authors declare no conflicts of interest.

## Data Availability

The data that support the findings of this study are available from the corresponding author upon reasonable request.
